# A Review of Research Progress in Microfluidic Bioseparation and Bioassay

**DOI:** 10.3390/mi15070893

**Published:** 2024-07-08

**Authors:** Heng Zhao, Yanyan Zhang, Dengxin Hua

**Affiliations:** Center for Lidar Remote Sensing Research, School of Mechanical and Precision Instrument Engineering, Xi’an University of Technology, Xi’an 710048, China.; hzhao@xaut.edu.cn (H.Z.); 13484422636@163.com (Y.Z.)

**Keywords:** microfluidics, active separation, passive separation, hybrid separation, bioseparation, bioassay, particles

## Abstract

With the rapid development of biotechnology, the importance of microfluidic bioseparation and bioassay in biomedicine, clinical diagnosis, and other fields has become increasingly prominent. Microfluidic technology, with its significant advantages of high throughput, automated operation, and low sample consumption, has brought new breakthroughs in the field of biological separation and bioassay. In this paper, the latest research progress in microfluidic technology in the field of bioseparation and bioassay is reviewed. Then, we focus on the methods of bioseparation including active separation, passive separation, and hybrid separation. At the same time, the latest research results of our group in particle separation are introduced. Finally, some application examples or methods for bioassay after particle separation are listed, and the current challenges and future prospects of bioseparation and bioassay are discussed.

## 1. Introduction

As a field that integrates science and engineering principles, microfluidics focuses on the behavior, manipulation, and control of fluids and particles at the microscale [1,2,3,4,5]. Since the early 1990s, with the innovative development of biocompatible materials and process mechanisms, microfluidic technology has been widely used in many fields including chemical synthesis, proteomics, single cell analysis, tissue engineering, environmental analysis, and medical diagnosis [6,7,8,9,10], Especially in the field of biological separation and bioassay, microfluidic technology has become a revolutionary tool to promote research progress in these two fields with its unique advantages [11,12,13,14,15,16,17,18].

In terms of bioseparation, microfluidic technology achieves the efficient, rapid, and accurate separation of target molecules in biological samples by accurately controlling the fluid flow and particle motion in microchannels, which greatly improves the separation efficiency and accuracy. A microfluidic system is an ideal device for biological particle separation [19] and exhibits a wide range of advantages including low sample usage, portability, relatively low manufacturing costs, improved sterility, rapid processing, and direct integration with downstream analysis components [20,21].

**Figure 1:**
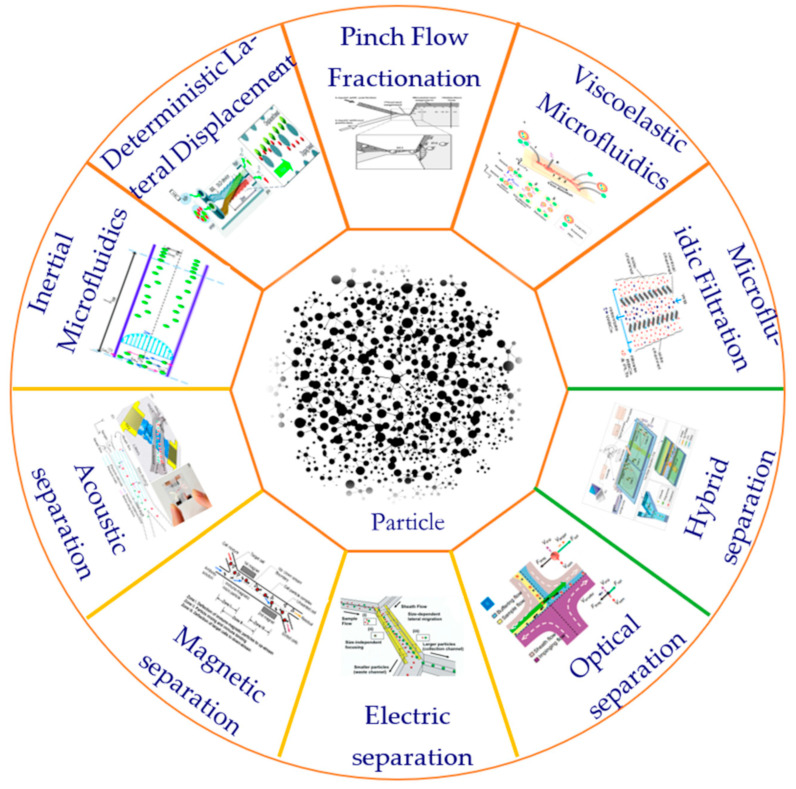
Commonly used particle separation methods.

At present, many techniques have been proposed and developed to manipulate particles in microfluidic systems. According to the source of the control force, these techniques can be divided into active and passive categories [22]. Active technology such as the use of magnetic fields [23], optical fields [24], electric fields [25], acoustic fields [26], and other external field forces can be used to directly control the movement of particles. Passive technology is completely dependent on the channel geometry or inherent fluid dynamics such as deterministic lateral displacement (DLD) [27], pinch flow separation (PFF) [28], inertial microfluidics [29], viscoelastic microfluidics [30], microfluidic filtration, etc. [31]. In addition, there is also a hybrid separation method combining active and passive techniques that shows significant advantages in the separation of biological particles. The method of particle separation is shown in Figure 1.

In the field of bioassay, the application of microfluidic technology provides a powerful tool for biomedical research and clinical diagnosis. Microfluidics is characterized by the study and manipulation of fluids at the submillimeter scale [32]. The combination of microfluidic technology and traditional biological detection methods can greatly promote the development of this field [33]. For example, microfluidic systems can reduce the size of the experiment and thus provide some useful functions. These include the ability to use a smaller number of samples and reagents, lower costs, and shorter analysis times [34]. After combining the microfluidic channel with functional units, the reaction, preparation, separation, and detection can be integrated into one chip [35]. If multiple reaction units are integrated together, high-throughput screening can be achieved [36]. Feedback control and sensing systems can also be integrated with microfluidic devices to manage the automation of on-chip processes [37].

In the field of bioseparation and bioassay, microfluidic technology has shown extraordinary importance. By applying microfluidic technology to bioseparation, we can achieve more accurate, efficient, and automated sample processing, which greatly improves the separation efficiency and accuracy. At the same time, in terms of bioassays, microfluidic technology provides a more sensitive and rapid detection method, which is helpful for the rapid diagnosis of diseases, the monitoring of environmental quality, and the screening of drugs.

So far, the methods of bioseparation have been summarized by many researchers. In order to further explore the application of microfluidic technology in bioseparation and bioassay, this paper reviews the latest progress of microfluidic systems in the field of biological separation, then discusses how they work, and summarizes the research undertaken by our group in particle separation. Finally, the research examples of further bioassays after biological particle separation are listed, and the challenges and future development directions of microfluidic technology in biological separation and bioassay are summarized.

## 2. Bioseparation Technology Based on Microfluidics

As an important branch of modern biotechnology, microfluidic-based bioseparation technology has been widely used in many fields. Based on the difference in manipulation force, this technology is classified into two types: passive and active methods. Passively relying on the physical properties of biological samples, separation is achieved by the specific structure in the microfluidic chip, which is easy to operate and does not damage the sample. In contrast, the active use of external force fields such as acoustic fields, magnetic fields, electric fields, and optical fields to achieve accurate separation has high flexibility and accuracy. In order to provide a comprehensive overview, a detailed table is provided that encapsulates basic parameters such as work force, separation markers, the merits and demerits of each method, and their applicability, as shown in Table 1.

### 2.1. Passive Microfluidic Bioseparation Technology

Passive microfluidic bioseparation technology mainly depends on the physical properties of biological samples (such as size, shape, density, etc.), and achieves separation through specific structures in microfluidic chips (such as microsieve, micropore, etc.). The advantage of this technique is that it is simple to operate, does not require an external power source, and can achieve the non-destructive separation of biological samples.

#### 2.1.1. Inertial Microfluidics

Inertia microfluidic technology, as a cutting-edge technology for biological separation using fluid inertia effects at microscales, has made significant progress in the biomedical field in recent years. In 2007, Carlo et al. proposed the concept of inertial microfluidics [38]. In inertial microfluidics, the flow inertia is significant (1 < *Re*< 100). Limited fluid inertia will produce two effects: inertial migration and secondary flow. Inertial migration is a phenomenon in which randomly dispersed particles migrate to a specific cross-sectional equilibrium position in a straight channel due to hydrodynamic forces, known as inertial lift. Based on the assumption that the particle size is much smaller than the channel size, the net inertial lift (*F_L_*) can be expressed as [39].

(1) $$F_{L}=\frac{\rho_{f}U^{2}d^{4}}{H^{2}}f_{L}\left(Re,x\right),$$

where *U* is the mean flow velocity, *H* is the hydraulic diameter, and *f_L_* is the dimensionless lift coefficient of the function of Reynolds number and normalized cross-section position (*x*).

**Figure 2:**
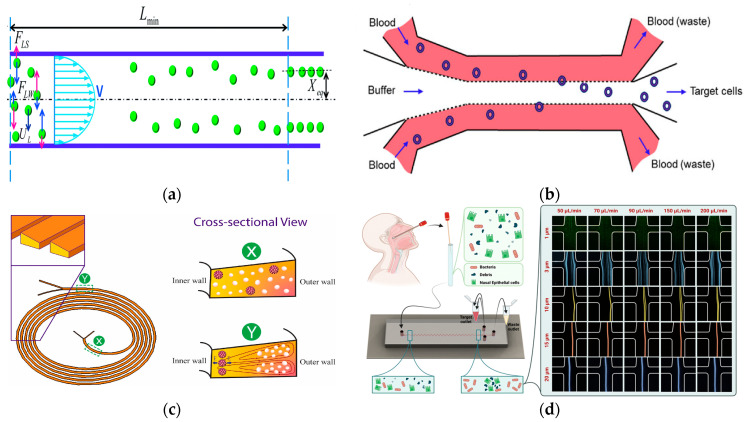
Separation method based on inertial microfluidics. (**a**) The lateral migration speed *U_L_* and minimum channel length for particle focusing *L*_min_. Reproduced with permission [41], copyright line © 2016 Royal Society of Chemistry. (**b**) Schematic of our size-based inertial microfluidic system used to isolate target cells from mouse blood. Reproduced with permission [42], copyright line © 2023 John Wiley and Sons. (**c**) Microfluidic devices with spiral configuration and trapezoidal cross-sections to separate CTCs from WBCs. Reproduced with permission [50], copyright line © 2022 Elsevier B.V. (**d**) Used for separating bacteria from epithelial cells and cell fragments in microfluidics. Reproduced with permission [53], copyright line © 2023 Royal Society of Chemistry.

Secondary flow is usually induced in a curved channel or a channel with a disturbed obstacle by the curvature or the fluid momentum mismatch between the center and the near-wall region in the obstacle. The balance between the secondary flow resistance (*F_D_*) and the inertial force (*F_L_*) determines the final focusing position and mode of the particles. When *F_L_* >> *F_D_*, the particles will focus on the equilibrium position. If *F_L_* ≪ *F_D_*, there will be no focusing phenomenon. In a straight channel, by balancing the net inertial lift and the Stokes drag, the transverse migration velocity (*U_L_*) and the minimum channel length (*L*_min_) of the particle can be obtained, which is the minimum length required for the particle to migrate to its inertial equilibrium position [40,41], as shown in Figure 2a.

In 2023, Macaraniag et al. successfully isolated breast cancer EO771 and PY230 cells in blood at a speed of 70 μL/min by using Newtonian sheath to flow between straight microchannel sample solutions (AR = 0.33) [42], as shown in Figure 2b. The sheath-assisted inertial separation of cells was also successfully demonstrated in spiral microchannels, where WBCs or MCF-7 cells were successfully separated at a blood flow rate of 2.4 mL/min and 1.2 mL/min, respectively [43,44]. Due to the cumulative effects of inertia and Dean force, the separation performance was gradually improved with the increase in channel length, both of which acted on the flowing sample in a size-dependent manner [45]. Contraction–dilatation microchannels were also used to separate other types of cells including T47D breast cancer cells in blood at a rate of 1 mL/min [46], and MCF-7 cells in blood at a rate of 750 μL/min [47]. Recently, a study using expansion–contraction microchannels achieved the size separation of MCF-7 cells and white blood cells at a flow rate of 7.5 mL/min [48].

Spiral microchannels have become a popular choice for size-based cell separation in inertial microfluidics due to their simple design, relatively long channel length, and the use of Dean flow. White blood cells have been successfully isolated from blood using spiral microchannels [49]. In 2023, Akbarnataj et al. proposed a new microfluidic device based on size-dependent cell sorting. The device had a trapezoidal cross-section and an elliptical spiral configuration, which achieved label-free and ultra-fast CTC enrichment, as shown in Figure 2c. The results showed that the separation efficiency of the device was the best when the flow rate was 2.5 mL/min, which was in good agreement with the numerical analysis results [50]. In 2023, Omrani et al. demonstrated a hydrodynamic-based passive, size-based, label-free microfluidic method using an unconventional (long-ring and U-turn combination) spiral microfluidic device to separate CTCs and WBCs at a flow rate of 1.7 mL/min, achieving both separation efficiency and purity above 90% [51].

In 2022, Bazaz et al. developed a zigzag microchannel for rigid inertial separation and enrichment, hereinafter referred to as Z-RISE. Experiments showed that Z-RISE could enrich white blood cells and their subtypes from diluted and lysed blood while consuming dead cells, fragments, and red blood cells [52]. In 2023, Shrestha et al. proposed a novel microfluidic channel with a zigzag structure to enhance the isolation and detection of human clinical nasal swab bacteria, as shown in Figure 2d. This microfluidic zigzag channel separated bacteria from epithelial cells and debris by focusing on size differences [53].

#### 2.1.2. Deterministic Lateral Displacement

In 2004, the deterministic lateral displacement (DLD) technique was proposed. Huang’s team accidentally discovered this phenomenon when looking for the asymmetric diffusion of DNA in the barrier array and applied it to the separation of 1 μm and 0.9 μm polystyrene microspheres, DNA separation, and red and white blood cell separation [54]. As a passive microfluidic separation technology, DLD has been widely used in the separation of various biological particles from blood cells to exosomes. This technique uses size, shape, and deformability as potential physical markers for separation. During the migration process, particles larger than the critical diameter (*D_c_*) move laterally, and the particles smaller than the critical diameter (*D_c_*) move forward in a zigzag manner, resulting in the separation of particles by size. Empirically, *D_c_* can be expressed as [55].

(2) $$D_{c}=1.4G\varepsilon^{0.48},$$

In the formula, *G* is the gap between the columns, and *ε* is the displacement fraction. Here, *ε = tan θ*, where *θ* is the cylindrical gradient. The DLD device could isolate the hard osteosarcoma MG-63 cells from bone marrow cells [56]. In addition, it was found that the circular column DLD could achieve the size-dependent separation of microorganisms and particles including 1 μL/h Streptococcus pneumoniae and 5.5 μL/min fungal spores as well as 30 μL/min Escherichia coli [57,58,59]. In 2022, Sherbaz et al. successfully isolated Escherichia coli from spherical microcells with a diameter of 450–500 nm at a flow rate of 50 μL/min, showing the versatility of DLD in cell separation [60].

**Figure 3:**
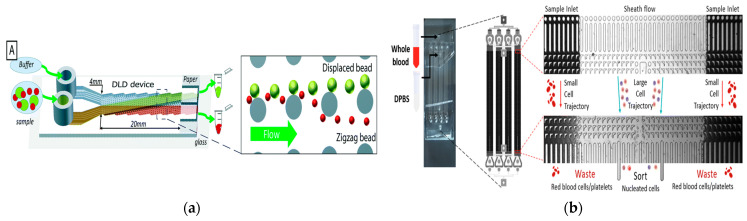
The separation method based on DLD. (**a**) The deterministic lateral displacement mechanism pushes the particles (green) larger than the critical size *D*_c_ from the sample flow to the co-flow buffer flow moving along the device. Smaller particles (red) remain in the sample stream. Reproduced with permission [61], copyright line © 2017 Royal Society of Chemistry. (**b**) Inverted L-shaped pillar arrays for separating nucleated cells from red blood cells and platelets. Reproduced with permission [65], copyright line © 2023 Royal Society of Chemistry.

In 2017, Tran et al. proposed a simple particle and cell separation scheme based on the control of fluid flow on the pattern surface to separate [61], as shown in Figure 3a. In 2020, in order to expand the dynamic range of separation granularity, Yin et al. proposed a new cascade multi-stage DLD structure and established a visual and robust mathematical parameter interaction model that had the potential to break through the minimum separation size obstacle of DLD separation in many applications and was also conducive to optimizing the overall equipment size [62]. In 2020, Bhattacharjee et al. proposed an asymmetric circular column DLD array to improve the efficiency of separating circulating tumor cells. The fluid channel in the structure also made the resistance of the whole chip smaller, and the simulated sorting purity at the outlet reached about 100% [63]. In 2023, Li et al. demonstrated the versatility of their microfluidic platform. They used a dual-array DLD device to successfully isolate DU145 prostate cancer cells from the blood. The separation was based on cell size and stiffness, and the flow rate was 35 μL/min [64]. In 2023, Zen et al. developed a DLD device with an inverted L-shaped array for separating blood cells according to size, as shown in Figure 3b. This design effectively isolated larger nucleated cells from smaller red blood cells and platelets [65].

#### 2.1.3. Pinch Flow Fractionation

Pinch flow fractionation (PFF) is a particle separation technology based on the principle of spatial effect [66]. The pinch flow separation structure is simple and consists of two finer input branch channels and a wider output channel. The separation principle is to pass the sample and buffer into two inlets, respectively, and squeeze the sample particles to a thin layer near the edge of the narrow channel by increasing the buffer flow rate. Due to the shrinkage effect, all particles focus on a fixed position, while particles of different sizes focus on different positions, and smaller particles are closer to the edge. The focusing difference is expanded by the characteristics of laminar flow to separate particles of different sizes.

**Figure 4:**
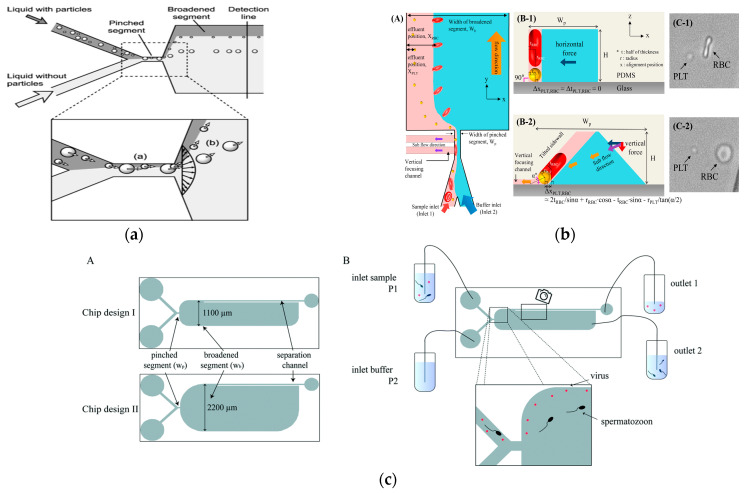
Separation method based on PFF. (**a**) Pinch flow fractionation schematic diagram. Reproduced with permission [67], copyright line © 2004 American Chemical Society. (**b**) Schematic diagram of separating discoid and spherical particles. Reproduced with permission [69], copyright line © 2017 Elsevier. (**c**) A microfluidic device for removing viruses in semen based on pinch separation. Reproduced with permission [72], copyright line © 2021 The Royal Society of Chemistry.

It is worth noting that in 2004, Yamada et al. proposed the concept of PFF, and they proved the size-based particle separation ability of PFF in microfluidics [67], as shown in Figure 4a. The distance difference (Δ*z*) between the separated particles of different sizes in the expansion region can be expressed as

(3) $$\Delta z=\frac{w_{p}\left(w_{p}-D_{p}\right)}{2w_{B}},$$

In addition to size, shape can also be used as a physical marker for the separation of PFF particles in microfluidics [68]. In 2017, Nho et al. reported the separation of disc-shaped and spherical particles in a more complex version of the PFF device, as shown in Figure 4b. The device had an inclined sidewall and a vertical focusing channel (called t-PFF-v). The separation of platelets and red blood cells was achieved, and the separation resolution of the t-PFF-v device was better than that of the previous classical PFF microchannels [69].

In general, the clamping region includes an extended region to promote cell separation. This was successfully demonstrated in various applications such as the use of tumbling behavior in sperm entrainment flow separation to separate it from red blood cells [70]. In 2019, Park et al. proposed the application of entrainment flow structure to the separation of fungi. Experiments showed that fungal spores could be separated from eukaryotic cells with a separation efficiency of more than 90% [71]. In 2021, Hamacher et al. proposed a microfluidic device based on entrainment flow for removing viruses in semen, as shown in Figure 4c. The system could extract sperm from the sperm injected with the virus at a recovery rate of 86%, and the virus removal rate was at least 86 ± 4% [72].

#### 2.1.4. Viscoelastic Microfluidics

Viscoelastic microfluidics achieve biological particle separation by adjusting the viscoelastic rheological properties of the carrier medium in the microchannel. The elastic force is generated due to the imbalance of normal stress in viscoelastic fluid flow. It is expressed as [73]:

(4) $$F_{E}=C_{eL}d^{3}\nabla N_{1},$$

**Figure 5:**
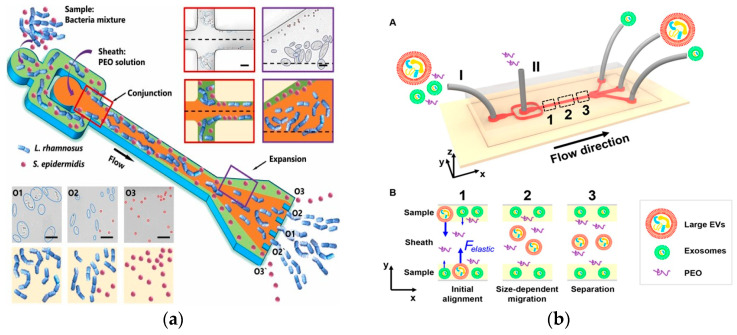
Separation method based on viscoelastic microfluidics. (**a**) Separation of *L. rhamnosus* and *S. epidermidis* using viscoelastic flows in a straight microchannel. Reproduced with permission [77], copyright line © 2023 Elsevier B.V. (**b**) (A) Schematic of the microfluidic chip for exosome separation from large EVs. Sample and sheath fluids containing a low concentration of PEO are introduced into the microchannel from the inlet (I) and the inlet (II), respectively. (B) Schematic illustration of the separation mechanism in viscoelastic microfluidics. Reproduced with permission [79], copyright line © 2017 American Chemical Society.

Among them, *C_eL_* is the dimensionless elastic lift coefficient and *N*_1_ is the normal stress difference. Subsequent studies demonstrated size-dependent separation. In 2019, Yuan et al. used a PEO solution to separate microalgae Chlorella from Bacillus subtilis at 1 μL/min [74]. In 2019, Lim et al. separated MCF-7 cells from white blood cells using an HA solution at 500 μL/min [75]. In 2019, Nam et al. used 100 μL/min to separate Candida albicans from white blood cells [76]. In 2023, a recent study by Zhang et al. achieved a size-based 19 μL/min bacterial separation using a PEO solution [77], as shown in Figure 5a. The microfluidic device was further used for separating E. coli single chains in PEO solution at a flow rate of 10 μL/min [78]. In 2017, Liu et al. proposed a viscoelastic-based microfluidic system to directly isolate exosomes from cell culture medium or serum in a continuous, size-dependent, and label-free manner, as shown in Figure 5b [79]. In 2023, Jia et al. developed stretchable microchannels for size-based microalgae cell separation with a cut-off size of 15–35 μm [80]. In 2022, Feng et al. used deformability for the physical labeling of cell separation [81]. In 2022, Zhang et al. used PEO solution to perform shape-based separation of E. coli less than 4 μm at a speed of 10 μL/min, which was the first application of this method in microfluidics [82].

In addition to the inertial force and elastic force, the secondary flow-induced Dean force also plays a role in the viscoelastic microfluidics of cell separation. For example, in 2021, a study by Yuan et al. used PEO solution to separate the length of bacteria *Cyanobacterial anabaena* (*C. anabaena*) at a rate of 40 μL/min in an expansion–contraction microchannel [83]. Under the combined action of Dean force and elastic inertia force, *C. anabaena* cells were divided into three subgroups: short-long (5~100 μm), medium-long (100~400 μm), and long (400~1000 μm). Similarly, in 2021, a study by Bilican used PEO medium to successfully separate Enterococcus faecalis from red blood cells in a size-dependent manner at a rate of 12 μL/min in a cascade contraction–expansion microchannel [84]. In spiral microchannels, in 2020, Zhou et al. demonstrated that viscoelastic flow could regulate particle focusing and separation. By using 10 ppm PEO solution at a flow rate of 160 μL/min, three different particle sizes of 3, 5, and 10 μm could be separated [85]. In 2020, Zhou et al. reviewed the physical and hydrodynamic characteristics of viscoelastic fluids. Three pairs of competing forces/effects were determined, which jointly controlled the viscoelastic migration. They also discussed the latest advances in migration dynamics, focusing position, numerical simulation, and viscoelastic microfluidic applications, and pointed out the remaining challenges [73].

#### 2.1.5. Microfluidic Filtration

Microfluidic filtration technology is an efficient micro-scale separation technology that uses a miniaturized ultrafiltration device in the microfluidic platform for particle separation. The core is to use a porous membrane as a separation medium to achieve the accurate separation of particles through precisely controlled microfluidic flow. When the fluid containing particles of different sizes passes through the porous membrane, particles smaller than the size of the membrane pores can pass through the membrane pores smoothly, while larger particles will be captured by the membrane pores, thus achieving the separation of particles [86].

**Figure 6:**
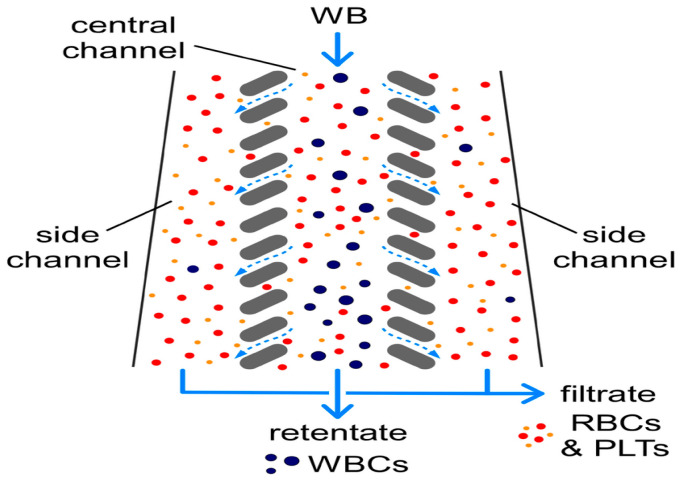
The separation of WBCs from RBCs and PLTs via controlled incremental filtration (CIF). Reproduced with permission [91], copyright © 2022, Springer Nature.

In 2008, Chen et al. used the principle of cross-flow filtration to separate white blood cells and red blood cells at a flow rate of 10 μL/min using column-type and weir-type microstructures. The separation efficiency of leukocytes by the cross-flow filtration microchip was about twice that of the terminal filtration microchip. In addition, plasma, white blood cells, and red blood cells could be separated and collected simultaneously at different outlets, with a multi-layer filtration barrier [87]. In 2022, Faustino et al. and Pinho et al. developed a multi-stage cross-flow filtration microfluidic device with a columnar microstructure that could simultaneously separate red blood cells and white blood cells from plasma and evaluate the deformability of cells [88,89]. In 2019, Mossige et al. also used the array microstructure to separate algae complexes by shape [90]. In 2022, Lezzar et al. described a new high-throughput microfluidic device based on controllable incremental filtration (CIF) technology [91], which can replace centrifuges for leukocyte separation. In flow mode, the efficiency of the CIF device for separating white blood cell was greater than 85%, as shown in Figure 6.

In 2023, Abhishek et al. proposed an innovative lymphocyte separation method that selectively binds multiple red blood cells to unwanted cells to form a rose structure, so the effective size of these cells is much larger than that of typical lymphocytes. Subsequently, the required lymphocytes can be easily separated from the nodular cells using a microfluidic size separation device based on controlled incremental filtration (CIF) [92]. In 2012, Cheow et al. introduced a microfabricated oblique sieve structure called a herringbone nanofilter array (HNA), which is formed by right-angle intersection of deep and shallow nanoslits with periodic patterns for the continuous flow of biomolecule concentration detection and the discrimination of analytes of different sizes. By combining fluorescent probes with samples and measuring their concentration effects, HNA technology can accurately analyze the concentration and size of biomolecules [93]. In 2017, Sung et al. proposed a nanofluidic device for the continuous monitoring of the purity and biological activity of biological products with high sensitivity, high resolution, and speed. Periodic and angled nanofilter arrays are used as molecular sieve structures for the continuous size-based analysis of biological products [94].

### 2.2. Active Microfluidic Bioseparation Technology

Although significant progress has been made in passive microfluidic bioseparation technology, it may be difficult to achieve ideal separation results in the separation process of some complex biological samples by only relying on the physical properties of biological samples. Active microfluidic bioseparation technology uses an external force field to manipulate and separate particles [95]. Acoustic, magnetic, electric, and optical fields are the most commonly used force fields in active microfluidic separation technology [19]. Due to its versatility and high controllability, active microfluidic separation technology has been customized to handle many submicron and nanoparticles including exosomes, bacteria, viruses, platelets, polystyrene, and gold nanoparticles [96,97,98,99,100,101,102,103,104,105]. The application of each manipulation force in the separation of submicron and nanobiological particles is described below.

#### 2.2.1. Biological Separation Technology Based on Acoustic Field

The integration of microfluidics and acoustic fluids allows for the precise separation of different types of biological particles such as cells, bacteria, or viruses. By applying an acoustic force to the fluid sample in the microchannel, these particles can be oriented to different regions according to their size, shape, or other characteristics.

**Figure 7:**
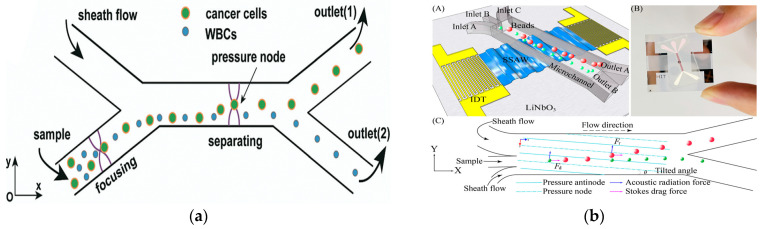
Separation method based on acoustic field. (**a**) The working principle of the device separation including the pre-centering zone and the separation zone, and the separation and collection of particles at the two outlets (1, 2) using the sheath flow. Reproduced with permission [106], copyright line © 2019 Royal Society of Chemistry. (**b**) (A) Schematic of the acoustofluidic separation chip using the taSSAW-based design. (B) object picture. (C) Schematic of the working mechanism of taSSAW-based particle separation as viewed from the top. In the hybrid acoustic pressure channel resonating area, larger particles (red dots) and smaller particles (green dots) are separated because of the difference in the vertical displacement. Reproduced with permission [111], copyright line © 2022 American Chemical Society.

In 2019, Wu et al. introduced an innovative glass-based acoustic streaming device for efficient, label-free, and non-invasive separation of circulating tumor cells (CTCs), as shown in Figure 7a. The experimental results showed that the average separation efficiency of the device for the mixed samples of MCF7 and HeLa cancer cells and white blood cells was about 91.5 ± 4.5%. The device has great potential in the application of cell separation and optical detection due to its excellent light transmittance [106]. In 2021, Zhang et al. showed a new type of omni-directional spiral surface acoustic wave (OSSAW) device for separating red blood cells and platelets from mouse blood with a purity of 93% and 84%, respectively [107]. In 2021, Simon et al., aiming at the defects of traditional acoustic streaming technology, proposed a simple acoustic device composed of an ultrasonic transducer driven by a pair of reconfigurable acoustic band-pass waveforms. The system could separate particles according to their size and mechanical properties, and was no longer limited to cell size. In addition, the device had good stability for flow changes [108]. In 2021, Li et al., for the first time, demonstrated label-free and high-throughput acoustic single-cell separation activated by characterizing multiple biophysical phenotypes [109]. In addition, the team also demonstrated a tunable acoustic flow control device for continuous particle separation based on various physical properties such as size, density, compressibility, and acoustic velocity [110]. In 2022, Han et al. proposed an acoustic flow separation chip that included a piezoelectric device that could produce a vertical saw with an inclined angle and a permanently bonded PDMS microchannel. By optimizing the control parameters, the separation of micron and submicron particles under different flux conditions was achieved [111], as shown in Figure 7b.

Exosomes are small extracellular vesicles secreted by cells containing parental cell components such as RNA, DNA, and proteins, which are essential for intercellular communication [112,113]. Researchers are paying more and more attention to the exosome separation technology of complex biological fluids. Unlike traditional ultracentrifugation and filtration methods containing multiple operation steps, the acoustic fluid-based method could be separated continuously, with less sample loss and lower possibility of structural damage, which provided a promising method for the separation of exosomes [114]. There have been many applications of acoustic streaming separation such as the separation of microorganisms and pollutants in environmental monitoring [115] as well as the separation and purification of food components such as proteins and enzymes [116], which have been widely used in the biological field.

The application of acoustic field separation technology in the biological field not only significantly improves the economic feasibility, but also shows strong scalable manufacturing potential. From an economic point of view, acoustic field separation technology is based on the operation of acoustic equipment. Compared with traditional technologies such as centrifuges or chromatographic columns, it has lower purchase and operation costs, simple maintenance, and helps to reduce long-term operating costs. In terms of extensible manufacturing, the sound field separation equipment adopts a modular design, which is convenient for expansion and upgrading according to production requirements. This flexibility enables the sound field separation technology to adapt to different scale production scenarios. In addition, acoustic field separation technology can also adjust and optimize parameters according to specific needs to achieve the accurate separation and purification of different biological components. It is worth mentioning that acoustic field separation technology can also be combined with other biological separation technologies (such as electric field separation, magnetic field separation, etc.) to form a multi-functional biological analysis system to cope with more complex biological separation tasks. Therefore, sound field separation technology has broad application prospects in the biological field due to its economic feasibility and scalable manufacturing potential.

#### 2.2.2. Biological Separation Technology Based on Magnetic Field

Magnetophoresis (MP) refers to the movement of particles relative to the fluid under the action of an external magnetic field. In 2009, Surenjav et al. introduced electromagnets into microfluidic platforms for droplet separation, which allowed magnetic separation to be widely used [117]. Positive MP can directly manipulate magnetic particles such as red blood cells or cells labeled with magnetic beads. Negative MP usually manipulates nonmagnetic particles in biocompatible magnetic media. With the advantage of its simple, low-cost, and non-contact, this technology has been widely used in the separation of submicron particles.

**Figure 8:**
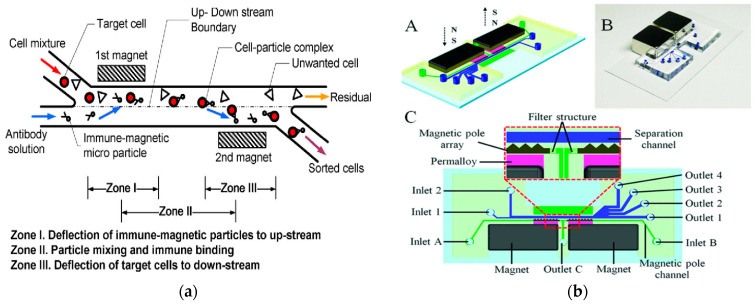
Separation method based on magnetic field. (**a**) The conceptual design of the new IMP cell sorter. Reproduced with permission [118], copyright © 2009, IEEE. (**b**) Schematic illustration and photograph of the microfluidic system. (A) Schematic drawing of the chip design; (B) Photograph of prototype chip; (C) Detailed chip design and enlarged view on the separation region. Reproduced with permission [122], copyright © 2021, IEEE.

In 2009, Kim et al. developed a new type of microimmuno-magnetic cell separating instrument for separating T lymphocytes from biological suspensions such as whole blood. By using two permanent magnets, the developed miniature IMP cell sorting machine could continuously and automatically separate target cells without a pre-labeling process [118], as shown in Figure 8a. In 2021, Zeng et al. developed a magnetic fluid device to separate 0.2 μm polystyrene particles and 1 μm polystyrene particles by negative MP [119]. In 2022, they modified the device to use the separation space between the sidewalls on both sides to increase the sample throughput. In the improved device, the sheath flow symmetrically focused the sample flow along both sides of the sidewall at the inlet. The high permeability alloy, the on-chip magnetic micropile array, and the permanent NdFeB magnet formed a pattern symmetrically on both sides of the separation channel so that the symmetrical ultra-high magnetic field repelled the nanoparticles to the center of the channel. They used this device to separate extracellular vesicles with a size of 30–200 nm from the cell culture supernatant, with a recovery rate of 85.8% and a purity of 80.45% [120]. In 2020, Liu et al. successfully separated exosome-like particles of 30–150 nm from biological samples using a similar iron fluid dynamics technique, with a recovery rate of 94.3% and a purity of 87.9%. This work used a four-pole configuration of four permanent magnets to provide high flux density and gradient [121].

In 2021, Lin et al. developed an interchanged microfluidic chip for the separation and sorting of immunomagnetic micro/nanoparticles-labeled leukocytes for biomedical applications. A three-in-three-out microfluidic chip was designed, achieving continuous horizontal and vertical separation [23]. In 2021, Zeng et al. proposed a high-resolution microfluidic magnetic separation system for the negative magneto-optical separation of nanoparticles, as shown in Figure 8b. The system generated an ultra-high gradient magnetic field greater than 10^5^ T/m in the separation channel by integrating two on-chip magnetic pole arrays. The microfluidic system was used to separate the mixed samples of 0.2 μm and 1 μm polystyrene particles. The recovery rate of 0.2 μm particles was 91.2% and the purity was 94.72%. This system was expected to be developed as a universal nanobiological sample separation and purification tool [122]. In 2020, Shamloo et al., in order to separate other heterogeneous cells from blood cells, designed a device for separating rare cells by using the negative magnetoconductivity effect on a rotating disk that was designed by numerical simulation. The simulation results showed that the model could quickly prepare samples. The exposure time of the cells in the magnetic medium was very short, so the function of the cells was not potentially threatened by the magnetic fluid [123].

Magnetic field separation technology has shown good economic feasibility in the biological field. Although the preparation cost of magnetic particles or magnetic labels may be high, their reusability can reduce the overall cost once they are prepared. In addition, magnetic field separation technology also has high separation efficiency and accuracy especially when dealing with complex biological samples, where its performance is particularly prominent. In addition, magnetic field separation technology also shows strong scalable manufacturing potential. With the continuous development of new magnetic materials, magnetic field separation technology has obtained more material choices. These new magnetic materials may have higher magnetization and stability, which means that the magnetic field separation technology can be applied in a wider range of biological separation applications. In addition, the design and production of magnetic field separation equipment also tend to be modular, which facilitates the expansion and upgrading of the equipment. By adjusting the equipment configuration, increasing the magnetic field strength, or optimizing the magnetic field distribution, the magnetic field separation technology can easily be adapted to the production requirements of different scales.

#### 2.2.3. Biological Separation Technology Based on Electric Field

Electric field-based bioseparation technologies include dielectrophoresis (DEP), induced charge electrodialysis (ICEO), and alternating current electrothermal flow (ACETF). These technologies can effectively realize the manipulation and separation of biological particles. These technologies produce specific forces or effects on biological particles through different electric field mechanisms to achieve the efficient control and accurate separation of biological particles.

DEP describes the particle motion caused by the non-uniform electric field acting on the induced dipole of the particle [124], the concept of which was first proposed in 1951 by Herbert Pohl [125]. When particles are exposed to a non-uniform electric field, they produce a polarization force. Under this force, microparticles can migrate to areas with high- or low-field density. Nowadays, DEP has become one of the most important technologies for the manipulation of micro/nanoparticles in microfluidics. Some recent studies have reported cases of particle separation based on DEP, showing the great potential of DEP in the fields of biotechnology and medicine.

**Figure 9:**
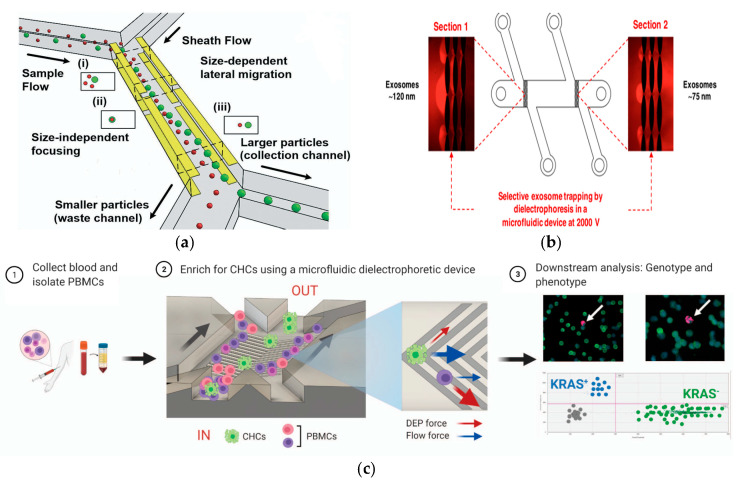
Separation method based on electric field. (**a**) Continuous and high-resolution size-based TDEP cell sorting. Reproduced with permission [130], copyright line © 2021 Royal Society of Chemistry. (**b**) The DC insulator-based dielectrophoresis (DC-iDEP) method can simultaneously capture and separate the size of exosomes. Reproduced with permission [131], copyright line © 2019 American Chemical Society. (**c**) Schematic representation of CHC enrichment from patient blood. Reproduced with permission [132], copyright line © 2021 Springer Nature.

In 2018 and 2020, Waheed et al. and Al-Ali et al. adopted an improved design form by using multiple sets of electrodes to achieve various operations such as the separation, focusing, switching, cleaning, and medium exchange of biological particles [126,127,128,129]. In 2021, Kung et al. displayed a microfluidic device that used tunnel dielectric electrophoresis (TDEP) to separate cells according to size differences, as shown in Figure 9a. The device could realize high-resolution three-dimensional operation of cell spatial position and could adjust the spatial position of cells of different sizes by adjusting the division of the electric field. This study provided a new idea for the accurate separation of cells [130]. In 2021, Huang et al. proposed a microfluidic chip that allowed a single cell flow line to drift gradually under medium electrophoresis. The isolated red blood cells were evaluated with the help of single cell impedance cytology. The results showed that the cells collected by the device were complete, and the method could solve the problem of ‘all-electric’ selective cell separation [124].

In 2019, Yoon et al. developed a periodically controlled positive DEP chip that could continuously isolate rare pathogens from the blood [100]. In 2019, Ayala-Mar et al. developed a DEP device based on a DC insulator that could capture and separate two different exosome subgroups according to size, as shown in Figure 9b. The device had two different channel sections that were filled with oval insulated columns to capture different populations of exosomes [131]. In 2021, Mira et al. reported a label-free DEP microfluidic separation platform for the high-throughput enrichment of circulating hybrid cells (CHCs) from human peripheral blood, as shown in Figure 9c. The experimental results showed that the system could deplete 96.5% of PBMCs, and the enrichment degree of CHCs was as high as 18.6 times. In clinical trials, 2 mL of blood from patients with pancreatic cancer could be processed in 1 h and a 75% enrichment rate could be achieved, establishing the potential of this method for non-invasive human pathological analysis in clinical practice [132].

ICEO is generated by an external field acting on its own induced diffusion charge in a thin boundary layer on a gate-polarizable surface in direct contact with the electrolyte [133]. In 2018, Sun et al. developed a switching technology based on asymmetric ICEO by using two pairs of driving electrodes and AC signal excitation. This technique can pre-focus the particles, control their lateral displacement, and then flexibly switch the particle flow to different downstream channels by configuring the driving electrode and reconstructing the external field signal [134]. ICEO-based micro-vortex technology has been successfully applied to effectively focus particles to a single outlet [135]. However, it is still a technical challenge to achieve the effective switching of ICEO-based particles between multiple outlets. In 2019, Sun et al. proposed a simplified multi-functional traffic control method that effectively combined DEP and alternating current heat flux to achieve continuous particle capture, switching, and classification [136].

Electric field separation technology has shown significant economic feasibility and scalable manufacturing potential in active microfluidic bioseparation technology. From the perspective of economic feasibility, the cost of electric field generation and regulation is relatively low. The use of electric fields can achieve rapid and efficient biological separation, which not only improves production efficiency, but also reduces the overall operating costs. In addition, in terms of scalable manufacturing potential, since the electric field module can achieve standardized design and production, it is convenient to construct biological separation systems of different scales and functions through the combination of modules. This modular design not only improves production efficiency, but also reduces the production costs, making electric field separation technology more competitive in large-scale production. In summary, microfluidic-based electric field separation technology not only has significant economic advantages, but also shows great potential in scalable manufacturing. In the future, with the continuous advancement of technology and the continuous expansion of application fields, electric field separation technology is expected to play a more important role in the field of biological separation.

#### 2.2.4. Biological Separation Technology Based on Optical Field

In 1970, scientists found that by using a focused laser beam, it was possible to maintain and move microscopic and sub-microscopic objects including atoms, nanoparticles, and droplets. This technique was later called optical tweezers. The optical force acting on the particle is the sum of the radiation scattering force and the gradient force [137]. The scattering force pushes the particle toward the direction of light, and the gradient force pulls the particle toward the direction with the highest intensity gradient. The induced optical force depends on the size, shape number, and refractive index of the particles. Optical tweezers are widely used in chemistry, physics, biology, medicine, and other fields [138,139,140,141].

**Figure 10:**
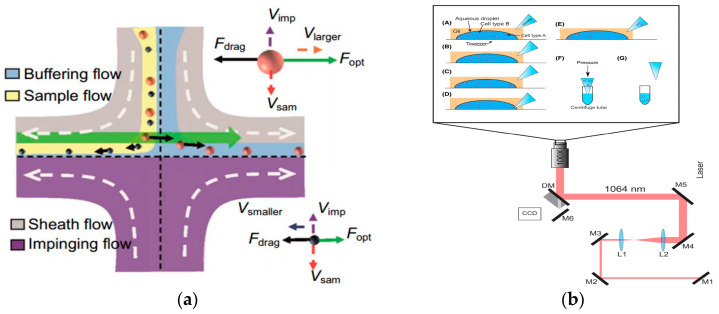
Separation method based on optical field. (**a**) Separating process of nanoparticles with different radii in fluid. Reproduced with permission [138], copyright line © 2016 American Chemical Society. (**b**) Schematic illustration of the optical tweezer cell separation and collection process. (A) An aqueous droplet containing a mixture of cells (blue and red circles) is fixed onto a glass coverslip in an oil background (hexadecane). A micropipette tip with a tip diameter of approximately 5–10 microns is positioned nearby. (B) Optical tweezers trap a target cell and position it near the edge of the droplet. (C) This process is repeated as many times as necessary to separate the desired number of cells. (D) The pipette tip is positioned at the edge of the droplet and the capillary force draws the cells up into the tip. (E–G) The pipette tip is removed from the oil solution and placed into a centrifuge tube containing lysis buffer. Reproduced with permission [144], copyright line © 2023 John Wiley and Sons.

In 2016, Wu et al. used optical and hydrodynamic forces to separate gold nanoparticles in a flow system and obtained high precision and considerable throughput. Experiments proved that 50 nm and 100 nm gold nanoparticles were separated from 100 nm and 100 nm gold nanoparticles [138], as shown in Figure 10a. In 2022, Shi et al. reviewed and discussed the manipulation of optical flow-controlled optical tweezers from the aspects of optical flow-controlled optical tweezer category, physical mechanism, and biomedical applications, and summarized the potential challenges of optical flow-controlled optical tweezer manipulation [142]. In 2022, Lv et al. proposed a label-free cell detection method based on a microfluidic chip. The cells were identified by measuring the scattering of the cells, and then the target cells were separated by optical tweezers. The whole process could realize the automatic identification and separation of the cells without any labeling and physical contact [143]. In 2024, Valle et al. reported a simplified and efficient cell transfer scheme using optical tweezers to efficiently separate sperm and white blood cells, which proved its advantages in reducing the sample preparation and shortening the capture time compared with traditional separation methods, which provided a new way for the cell separation of different forms [144], as shown in Figure 10b.

Optical field separation technology shows significant economic feasibility and scalable manufacturing potential in active microfluidic bioseparation technology. From the perspective of economic feasibility, optical field separation technology usually does not require the use of expensive chemical reagents or complex equipment. Compared with traditional biological separation methods, optical field separation technology can achieve the efficient and precise control of biological particles by accurately controlling the distribution and intensity of the optical field without complex pretreatment steps or subsequent processing procedures, thus saving time and resources. In addition, the scalable manufacturing potential is another important advantage of optical field separation technology. Optical field separation technology can easily be integrated into microfluidic chips to achieve high-throughput and automated biological separation. In addition, optical field separation technology can also be combined with other microfluidic technologies such as electric fields, magnetic fields, etc. to form a multi-field coupled bioseparation system, which further improves the efficiency and accuracy of bioseparation. This scalable manufacturing potential indicates that optical field bioseparation technology has broad application prospects and can play an important role in biomedicine, bioengineering, drug development, and other fields.

### 2.3. Research Progress of Hybrid Separation Technology

In microfluidic bioseparation technology, passive and active technologies have their own unique advantages. Passive separation technology has the advantages of high throughput and rapid efficiency due to its simple structure design and operation process. The disadvantage is that the resolution and separation purity are relatively low. On the other hand, active separation technology has the advantages of high purity and high resolution, but the disadvantage is that it requires the intervention of an external force field. In light of the respective advantages and disadvantages of active and passive separation technologies, their combination has become a potential solution to meet the needs of different application fields.

#### 2.3.1. Active–Passive Hybrid Separation Technology

In 2017, Yan et al. discussed the integration of active and passive microfluidic technology against the background of continuous cell separation in microfluidic systems [145]. In 2022, Al-Ali et al. reviewed the latest progress in the field of hybrid devices based on DEP and described the dielectric–passive and dielectric–active hybrid methods [146]. In 2023, Hettiarachchi et al. summarized the principle of traditional techniques for manipulating nanoparticles. Microfluidic technology was divided into passive, active, and hybrid. The physical principle, device design, working mechanism, and application of each technology were described in detail [147]. In 2024, Zhang et al. reviewed the wide application of passive microfluidic technology in cell separation including single and hybrid microfluidic separation methods [148]. In 2024, Ebrahimi et al. reviewed the development of biomolecular separation methods in the “chip laboratory” in the past decade, covering passive separation, active separation, and mixed separation techniques, focusing on the application of comprehensive complex separation methods [149].

**Figure 11:**
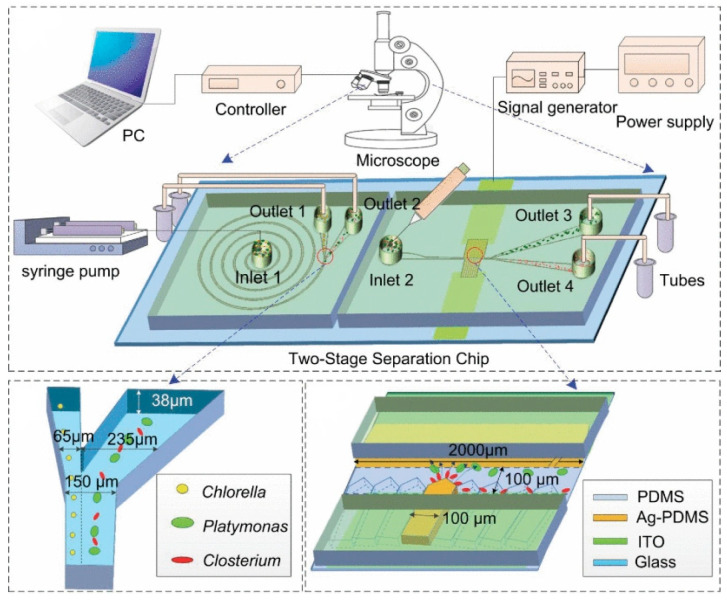
Active–passive hybrid separation method. A two-stage microfluidic separation chip combining inertia and DEP force was developed. Reproduced with permission [150], copyright line © 2020 IEEE.

In 2020, Wang et al. designed a two-stage microfluidic separation chip combining inertia and DEP force. As shown in Figure 11, it could achieve the rapid and accurate separation of three common microalgae cells (Platymonas, Closterium, and Chlorella) with a separation efficiency of more than 90% [150]. In 2022, Li et al. reported a new method combining a three-dimensional sidewall electrode and a contraction/expansion (CEA) structure to continuously separate particles of three different sizes or dielectric properties. Particle separation was achieved by the interaction of DEP force and inertial force. Due to the effect of inertial force, the CEA channel could separate particles of different sizes and enhanced the non-uniformity of the electric field. The three-dimensional electrode produced a non-uniform electric field at the same height as the channel, which increased the range of action of the DEP force, and the separation rate of PS particles was above 90%, which provides a new method for separating biological particles [151]. In addition, in 2023, Islam et al. proposed a new method that combined curved contraction–expansion (CE) channels with DEP and inertial microfluidics to separate circulating tumor cells (CTCs) and white blood cells (WBCs). This label-free continuous separation method used the dielectric properties and size changes of cells to separate CTCs and WBCs [152].

In 2020, Xu et al. separated free extracellular DNA by MP. Free DNAs were labeled with magnetic particles and then washed with appropriate methods. Molecular separation was achieved by combining MP and viscoelasticity [153]. In 2020, Zhang et al. proposed a magnetically driven deterministic lateral displacement (m-DLD) microfluidic device. A permanent magnet was placed at the outlet of the microchannel to generate the driving force. The m-DLD device could be used for antibody recognition and separation in an antibody mixed solution [154]. In 2018, Ros et al. successfully separated submicron biological particles using the integration of insulator-based DEP (iDEP) and nonlinear microfluidic column arrays, proving that the integration of DEP-DLD manipulates biological particles. By incorporating DEP into the DLD system, the combined separation force allowed for a wider column array, thereby improving the throughput and reducing channel blockage [155].

The combination of passive and active microfluidic bioseparation technology can give full play to the advantages of the two technologies and achieve more efficient and accurate bioseparation. For example, the channel design of introducing passive technology into active technology can improve the separation efficiency; the introduction of active force field manipulation in passive technology can improve the accuracy and controllability of separation. The combination of passive and active bioseparation technology has broad application prospects in the fields of cell therapy, drug screening, disease diagnosis, and so on. In the future, with the continuous development and optimization of microfluidic technology, the combination of passive and active bioseparation technology will show its unique value in more fields.

#### 2.3.2. Research Progress of the Center for Lidar Remote Sensing Research at Xi’an University of Technology

In the field of particle separation, we have maintained a high degree of attention. It is not only discussed and studied at the theoretical level, but also verified and analyzed at the experimental level. Among them, inertial separation and optical separation are the particle separation methods we have focused on.

**Figure 12:**
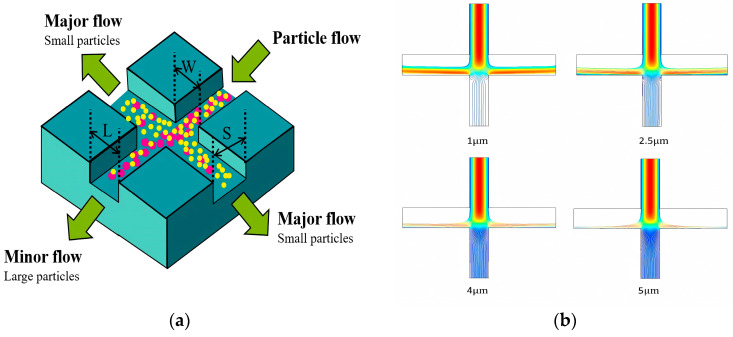
Inertial separation schematic diagram. (**a**) The schematic diagram of the inertial separation chip structure. The powder ball represents the particles with large particle size, and the yellow ball represents the particles with small particle size. (**b**) The trajectory of particles with different particle sizes.

Inertial separation technology is a separation technology based on the inertial difference of particles in the flow medium. This technology effectively realizes the efficient separation of particles of different sizes by accurately designing the specific flow field structure and parameters. At present, remarkable research results have been achieved in the field of inertial separation technology. As shown in Figure 12a, we designed a cross-shaped inertial separation structure. By analyzing the influence of key parameters (channel width, flow rate, particle size, etc.) on the separation effect, the optimal parameter combination was found. The structure could be separated based on the size of the particles. The particles with a larger particle size mainly flowed into the small flow channel, while the particles with a smaller particle size flowed into the main flow channel. By changing the size of the particles, as shown in Figure 12b, it could be clearly observed that as the particle size increased, the particles were more inclined to flow into the small flow channel. This further verified the effectiveness and accuracy of the inertial sorting structure [156,157,158].

**Figure 13:**
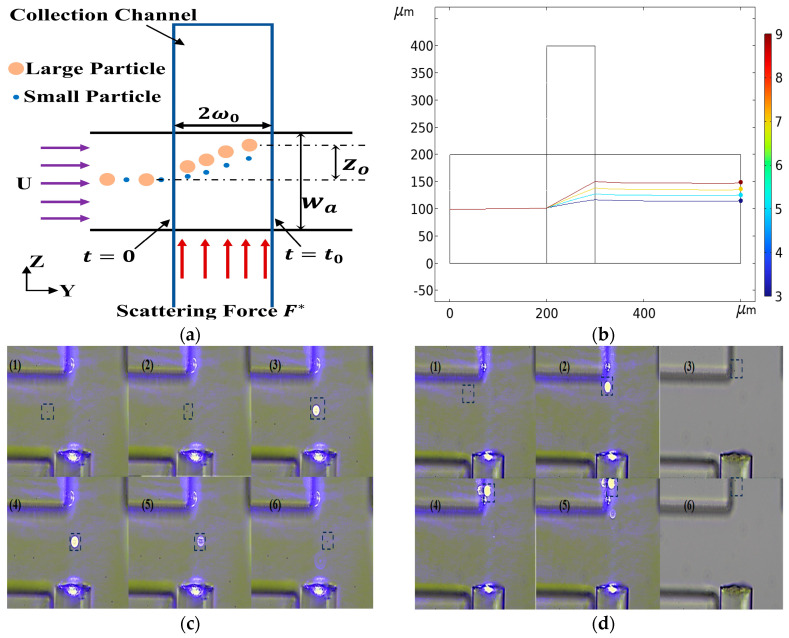
Optical separation schematic diagram. (**a**) Optical separation chip structure diagram, F^*^ is the optical scattering force. (**b**) Particle trajectories with different particle sizes. (**c**) The trajectory of 2 μm particles, (1)–(6) is the trajectory of 2 μm particles at different times, highlighted with a box. (**d**) The trajectory of 5 μm particles, (1)–(6) is the trajectory of 5 μm particles at different times, highlighted with a box.

After successfully realizing inertial separation technology based on particle size difference, we further explored the potential of optical separation technology. Optical separation technology uses the difference of particles with different particle sizes when they are subjected to light radiation force to separate. This principle provides us with another effective particle separation method. For this technology, we not only deeply analyzed its separation theory, but also built an experimental platform to verify its feasibility. As shown in Figure 13a, a T-shaped optical separation chip structure was designed. The trajectories of particles with different particle sizes were obtained by numerical calculation, as shown in Figure 13b. The trajectory of 2 μm particles passing through the laser irradiation area was verified by implementation (Figure 13c) as was the trajectory of the 5 μm particles when passing through the laser irradiation area (Figure 13d). The results showed that the difference of particle size led to the difference of optical force. Specifically, the larger the particle size, the greater the optical force. Based on this principle, we successfully achieved the separation of particles with different particle sizes.

Inertial separation and optical separation have their own unique advantages, so they are suitable for different application scenarios. Through our in-depth study of both technologies, the efficiency and accuracy of particle separation have been significantly improved, which has made a positive contribution to the development of the field of particle separation. In subsequent research, we hope to further explore the application of the combination of both of these technologies in the field of biological particle separation. Through continuous research and innovation, the efficient and accurate separation of biological particles can be achieved to provide strong technical support for the development of biomedicine, bioengineering, and biopharmaceuticals.

## 3. Microfluidic Bioassay Technology

With the continuous development and application of biotechnology, bioassays after bioseparation are increasingly attracting attention. This increase in attention is not only due to the promotion of technological progress, but also because the bioassay has shown its indispensable importance in many fields. Bioassays can accurately identify and analyze specific components or biomarkers isolated from biological samples and provide a scientific basis for the early diagnosis of diseases, the formulation of treatment plans, and the evaluation of drug efficacy. In addition, bioassays also show broad application prospects in food safety, environmental monitoring, and other fields. However, traditional bioassay methods still face certain challenges in terms of sensitivity, speed, and automation. Therefore, microfluidic-based bioassay technology has emerged accordingly. Its unique advantages make bioassays more efficient and accurate, greatly improving the sensitivity and speed of bioassays. It is expected that it can make greater contributions to human health, social development, and environmental protection.

**Figure 14:**
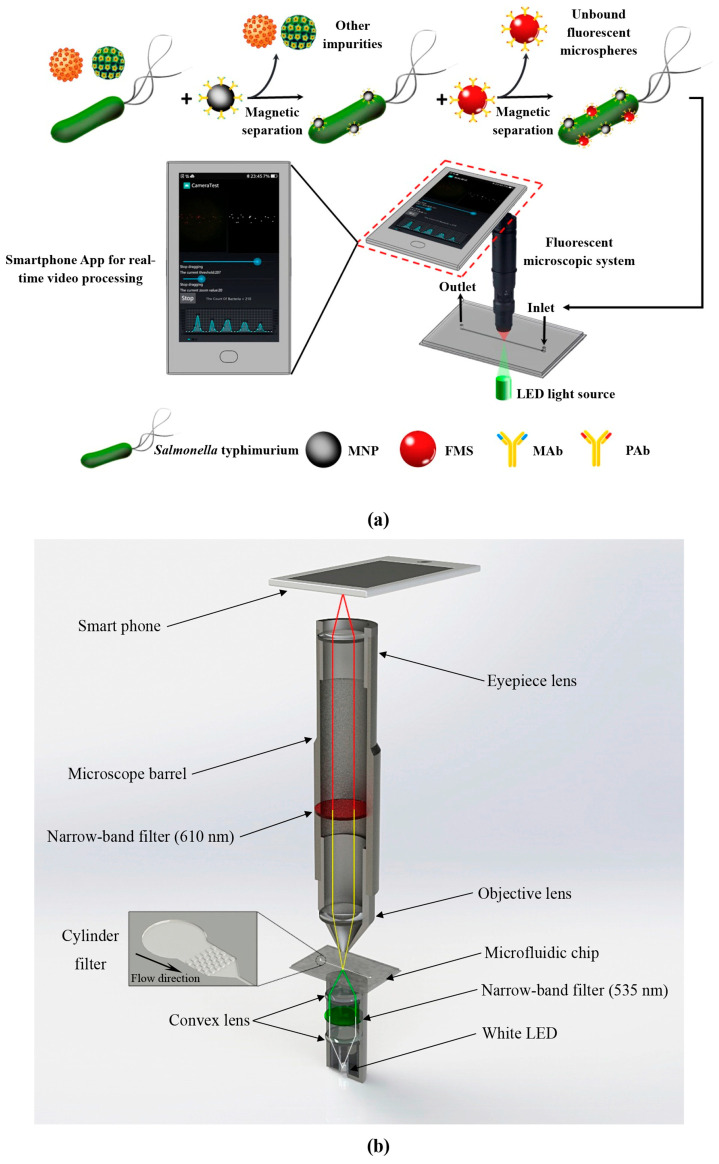
The schematic diagram of microfluidic detection process for Salmonella. (**a**) The principle of the microfluidic biosensor for online, rapid, and sensitive detection of *Salmonella typhimurium*. (**b**) The structure of the fluorescence microscopy system based on a smartphone. Reproduced with permission [159], copyright line © 2019 Elsevier B.V.

In 2019, Wang et al. developed a microfluidic biosensor for the online sensitive detection of Salmonella based on immunomagnetic separation, fluorescence labeling, and smartphone video processing [159]. The target bacteria were specifically separated and efficiently concentrated by immunomagnetic nanoparticles to form magnetic bacteria, and then the magnetic bacteria were labeled with immunofluorescence microspheres to form fluorescent bacteria. Then, the fluorescent bacteria were continuously injected into the microfluidic chip of the smartphone-based fluorescence microscopy system, and the fluorescent spots were counted online using the smartphone app based on the frame difference algorithm to obtain the number of target bacteria for the online, rapid, and sensitive detection of *Salmonella typhimurium*, as shown in Figure 14. Under the optimal conditions, the sensor could detect *Salmonella typhimurium* as low as 58 CFU/mL within 2 h, which has great application potential in the online monitoring of foodborne pathogens.

At present, the performance of this biosensor is primarily constrained by the video processing speed and image capture quality. To enhance its sensitivity, brighter fluorescent materials can be employed to label bacteria, thereby amplifying the fluorescent signal. Additionally, utilizing smartphones or devices with higher-performance specifications such as faster CPU speeds and superior camera resolutions would enable more accurate and rapid counting of fluorescent bacteria. Furthermore, integrating this biosensor technology with microfluidic techniques holds the promise of achieving accurate on-site detection of individual bacteria.

**Figure 15:**
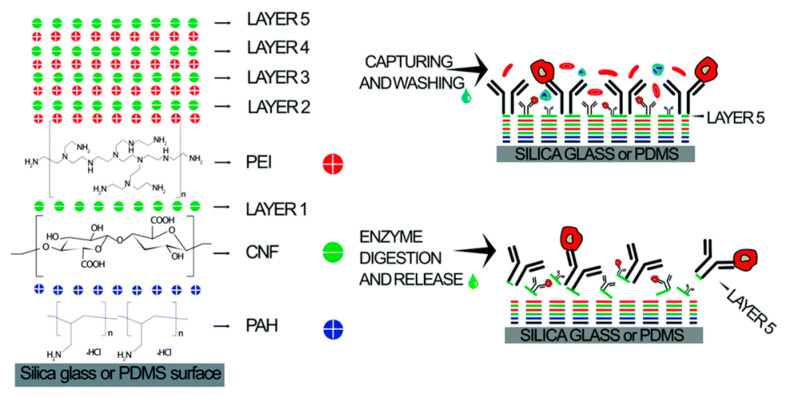
Schematics of LbL CNF assembly for affinity cell capture and enzymatic release. Reproduced with permission [160], copyright line © 2020 Royal Society of Chemistry.

It is particularly noteworthy that surface modification plays a crucial role in the sorting of subpopulations of cells by affecting cell adhesion, migration, and other surface interactions. In 2020, Kumar et al. developed a microfluidic chip modified with multilayer CNF components for effective immune affinity cell capture and enzymatic release, as shown in Figure 15. The combination of CNF nanostructure characteristics and microfluidic flow cells provides great potential for cell capture [160].

The device is composed of five layers of CNF and coated with the anti-EpCAM antibody, which can directly capture HCT 116 cells in whole blood. In addition, the capture efficiency is as high as 97% at 41.25 μL/min. This optimized microfluidic device provides a powerful tool for the accurate and sensitive separation and enrichment of rare cells, helping to expand and improve clinical diagnostic applications, especially in cancer screening and diagnosis.

In order to ensure that the cell capture process in the microfluidic device can be effectively monitored, a fluorescence microscope was used in the optimization experiment. In addition, in order to more fully characterize the cell, capture, and release processes, in addition to the subsequent immunocytochemistry, the cells were pre-stained with the cell-penetrating calmodulin AM activity dye, thereby enhancing the visibility of the cells under the microscope.

**Figure 16:**
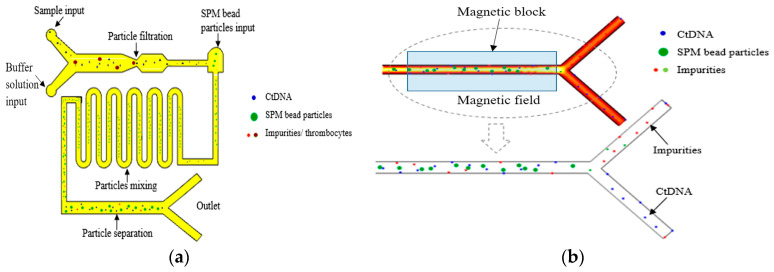
Magnetic field-assisted separation of ctDNA from SPM particles. (**a**) Top view of the microfluidic channel: filtration of ctDNA from thrombocytes of plasma input, mixing of ctDNA, and SPM bead particles in curved microchannel and binding of ctDNA into SPM bead particles for further separation by magnetic field. (**b**) Separation of ctDNA from SPM bead particles through the magnetic field formed by the magnetic block. Reproduced with permission [161], copyright line © 2021 Elsevier B.V.

In 2021, Balakrishnan et al. simulated the extraction and separation of circulating tumor DNA (ctDNA) in the plasma of Stage I and Stage II cancer patients using superparamagnetic (SPM) microsphere particles on a microfluidic platform to achieve early and effective cancer detection [161], as shown in Figure 16. The extraction of ctDNA was based on microfiltration and particle size operations to filter some impurities and platelet plasma. The typical size of ctDNA is a width of 2.6 nm and a length of 100 bp long. The thrombus cells are biconvex disk-like structures with a diameter of 2 μm to 3 μm, while the SPM beads are spherical particles with a diameter of 1 μm.

First, ctDNA could be separated from platelets before entering the next part of the microchannel. Second, an inlet with the width of 1.5 μm was designed at the other end of the microchannel, especially for the input of SPM particles. The ctDNA and SPM particles moved to the second part of the curved microchannel, which was designed for the effective mixing of particles. When the ctDNA was fully hybrid with the SPM beads, it could be effectively attached or absorbed to the SPM beads. When the solution was moved to the third part of the microfluidic channel, a permanent magnet was used to desorb or separate ctDNA from the SPM particles. The SPM particles were separated from ctDNA under the action of the magnetic field, and the SPM particles were fixed by a permanent magnet. The ctDNA could be collected in the outlet channel.

Under the action of a magnetic field, the method of using SPM particles to separate ctDNA not only had significant technical advantages, but also provided an extremely favorable and convenient rapid analysis method for the early detection of cancer. The results showed that ctDNA was effectively separated from an average of 5.7 ng per 10 μL of plasma input from patients with Stage I and Stage II cancer. This result not only demonstrates the great potential of SPM particles in the field of ctDNA separation, but also provides strong technical support for early screening and diagnosis of cancer.

**Figure 17:**
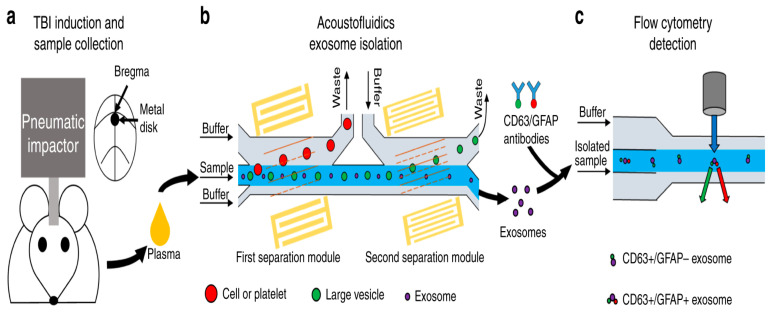
A schematic diagram of the process of detecting TBI exosome biomarkers from animal model blood. (**a**) Mouse brain injury model induced by pneumatic impactor. (**b**) Mouse plasma samples processed by acoustic flow chip. (**c**) Fluorescence labeling and flow cytometry detection. Reproduced with permission [162], copyright line © 2021 Springer Nature.

In 2021, Wang et al. used the combination of microfluidics and acoustics to separate biological particles according to the difference in particle size and acoustic characteristics, and successfully separated exosomes from the plasma samples of mice after traumatic brain injury. The acoustic fluid separation eliminated the interference of other blood components, which made it possible to detect exosome biomarkers of traumatic brain injury (TBI) by flow cytometry [162], as shown in Figure 17.

When the sample was processed using an acoustic fluid exosome separation chip, the particles in the sample were effectively separated according to their size differences when flowing through these devices. This separation technique could remove larger cells, platelets, and extracellular vesicles (such as apoptotic bodies and microbubbles) other than exosomes. The driving force behind this size-based separation came from the acoustic field generated by the integrated devices (IDTs) around the channel, which induced the acoustic radiation force to achieve accurate particle separation. 

Moreover, the separated samples were stained with fluorescently labeled anti-CD63 and anti-GFAP antibodies, and CD63+/GFAP+ was detected by flow cytometry. After TBI treatment, the number of GFAP+/CD63+ exosomes in plasma increased with time. By accurately detecting the number of exosomes, we can understand the situation of TBI patients in time to provide clinicians with an important reference for the current pathophysiological state of patients and help them develop more accurate treatment strategies. This detection method is not only fast and accurate, but also has high specificity and sensitivity, which provides an important basis for optimizing the treatment plan.

## 4. Conclusions

With the rapid development of biotechnology, microfluidic bioseparation and bioassay technologies have shown great potential and value in the fields of biomedicine and clinical diagnosis. Microfluidic technology has become an important breakthrough in the field of bioseparation and bioassay due to its unique advantages of high throughput, automated operation, and low sample consumption. In the study of bioseparation methods, we deeply discussed various technologies such as passive separation, active separation, and hybrid separation. Passive separation technology does not need to apply an external force field, but it is more dependent on the hydrodynamic effects and channel structure, and these factors may not fully meet the needs of efficient separation. In order to achieve effective passive separation, the channel design is often required to be very fine and complex. This includes considering the size, shape, curvature, surface properties, and other factors of the channel to ensure that the fluid can flow in the channel in the expected way and achieve separation. This high design complexity increases the difficulty and cost of manufacturing. Active separation technology can usually achieve the separation of biological samples faster and more effectively by applying an external force field. At present, the development of active separation technology is inseparable from the realization of more refined separation in biomedicine. However, the external position equipment used for active separation is relatively expensive, and the number of particles that can be processed at the same time may be limited, which affects the overall flux and processing efficiency.

In addition, our group also achieved the most significant and latest research results in particle separation. Through the theoretical research and experimental verification of inertial separation and optical separation, in order to achieve bioseparation more quickly and accurately, it is necessary to focus more on the field of hybrid technology combining active and passive separation. First, preliminary and rough particle separation are realized by using the natural physical process (i.e., passive separation technology), and then the external force or field (i.e., active separation technology) is introduced to achieve a more accurate and detailed separation and control of the target particles. With the deepening understanding of these two separation mechanisms, innovative hybrid separation methods have emerged. These methods skillfully combine natural laws with manual intervention, showing greater flexibility. Therefore, in the field of separation technology in the future, mixed separation technology will undoubtedly become a new hot spot in research and application. We look forward to promoting the development of hybrid separation technology to a more intelligent, efficient, and environmentally friendly direction by continuously optimizing and integrating existing technologies and exploring more unknown possibilities to meet the increasingly complex separation needs.

However, there are still some challenges in the field of bioseparation and bioassay such as improving the separation efficiency, reducing the detection costs, and achieving higher sensitivity and specificity. In order to overcome these challenges, future research needs to continue to explore new microfluidic technologies and materials as well as cross-integration with other disciplines to promote the further development and innovation of bioseparation and bioassay technologies. With the continuous progress of microfluidic technology and the continuous expansion of application fields, we have reason to believe that bioseparation and bioassay technology will play a more important role in biomedicine, clinical diagnosis, and other fields, and make greater contributions to human health.

## Figures and Tables

**Table 1:** Summary of typical techniques for bioseparation.

Technique	Principle	Separation Markers	Merits	Demerits
Inertial microfluidics	Inertial lift and Dean drag forces	Size, shape and stiffness	High throughput, simple	Strict design rules
Deterministic lateral displacement	Particles displace differentially in tilted pillar array	Size, shape, and stiffness	Simple, easy to operate	Clogging and fouling
Pinched flow fractionation	Pinching particles with hydrodynamic flows	Size and shape	Simple, higher separation	Low resolution
Viscoelastic microfluidics	Elastic force due to imbalance of normal stresses in a viscoelastic fluid	Size, shape, and stiffness	Simple, higher separation	Need for viscoelastic
Microfluidic filtration	Nanomembrane in microfluidics	Size, shape, and stiffness	High separation efficiency	Membrane clogging
Acoustic field separation	Acoustic force	Size, density, compressibility	Label free, biocompatible, contactless	Technical sophistication
Magnetic field separation	Magnetic force	Size, magnetic properties	High throughput, contactless	Time consuming and labor- intensive sample preparation
Electrostatic field separation	Electrostatic force	Size, electric polarizability	High separation efficiency	Induces thermal energy, electrochemical reaction
Optical field separation	Optical radiation scattering and gradient force	Refractive index, size	High separation efficiency	Low throughput, induces thermal energy, affects biocompatibility

## Data Availability

Data sharing not applicable.
